# Dietary Polyunsaturated Fatty Acids Regulate Dendritic Cell Function via Nrf2-dependent Control of Ferroptosis

**DOI:** 10.21203/rs.3.rs-7983397/v1

**Published:** 2025-11-19

**Authors:** Deepika Awasthi, Erin K. McMinn, Camilla Salvagno, Sung-Min Hwang, Tito A. Sandoval, Chang-Suk Chae, Wiam A. M. Madani, Jacqueline Crater, Kaushik Sen, Daniel Min, Alexander Emmanuelli, Chen Tan, Andrew J. Dannenberg, Diana K. Morales, Evelyn Cantillo, Eloise Chapman-Davis, William Z. Zhang, Suzanne M. Cloonan, Juan R. Cubillos-Ruiz

**Affiliations:** 1 Department of Obstetrics and Gynecology, Weill Cornell Medicine. New York, NY 10065, USA.; 2 Sandra and Edward Meyer Cancer Center, Weill Cornell Medicine. New York, NY 10065, USA.; 3 Weill Cornell Graduate School of Medical Sciences. New York, NY 10065. USA.; 4 Division of Pulmonary and Critical Care Medicine, Department of Medicine, Weill Cornell Medicine. New York, NY 10065, USA; 5 School of Medicine, Trinity Biomedical Sciences Institute, Trinity College. Dublin, Ireland

## Abstract

Dendritic cells (DCs) orchestrate adaptive immune responses to pathogens and tumors, yet how dietary lipids influence DC metabolism and function remains largely unexplored. Here we show that dietary polyunsaturated fatty acids (PUFAs) govern DC activity via Nuclear factor erythroid 2–like 2 (Nrf2)–dependent control of ferroptosis. In mice, an n-6 PUFA–enriched diet suppressed DC Nrf2 signaling, depleted glutathione, and induced lipid peroxidation and ferroptosis, thereby compromising antigen presentation. By contrast, dietary n-3 PUFAs enhanced Nrf2 signaling and redox homeostasis, preserving DC integrity and T cell priming. Pharmacologic Nrf2 activation or ferroptosis inhibition restored the function of DCs from n-6 PUFA–fed mice. Notably, adoptive immunotherapy with DCs conditioned by a diet rich in n-3 PUFAs—but not n-6 PUFAs—elicited durable, T cell–dependent control of metastatic ovarian cancer. These findings identify dietary PUFAs as key modulators of the Nrf2–glutathione–ferroptosis axis in DCs and reveal a redox-sensitive metabolic checkpoint that can be leveraged to improve cancer immunotherapy.

## Introduction

Polyunsaturated fatty acids (PUFAs) are essential for maintaining cellular homeostasis^1^. They serve as structural components of cell membranes, regulate membrane fluidity, function as an energy source, and act as precursors for signaling molecules involved in inflammation and immune regulation^2^. Since mammals lack the enzymatic capability to introduce double bonds beyond carbon-9 in fatty acid chains^3,4^, humans must obtain n-3 and n-6 PUFAs from dietary sources. The n-3 family—comprising α-linolenic acid (ALA), eicosapentaenoic acid (EPA), and docosahexaenoic acid (DHA)—is found predominantly in flaxseed, walnuts, marine phytoplankton, and fatty fish such as mackerel, salmon, and menhaden^5^. The n-6 family—including linoleic acid (LA), dihomo-γ-linolenic acid (DGLA), and arachidonic acid (AA)—is sourced primarily from vegetable oils (e.g., soybean, corn, sunflower) and animal products^5^. Of note, modern Western diets are disproportionately enriched in n-6 PUFAs due to widespread consumption of vegetable oils and processed foods, leading to a dietary imbalance that has been linked to increased inflammation and oxidative stress^6,7^. Despite extensive research on the metabolic and inflammatory consequences of n-6 versus n-3 PUFAs^4,8–10^, how these diet-derived lipids impact immune cell function remains largely unexplored.

Dendritic cells (DCs) are professional antigen-presenting cells (APCs) that play a pivotal role in bridging innate and adaptive immune responses^11^. By capturing, processing, and presenting antigens, DCs direct the activation of naïve T cells, making them essential for the initiation and maintenance of immunity to pathogens and tumors^12^. How nutritional and metabolic cues impact DC function and fate remains poorly understood. Indeed, while PUFAs have been shown to alter some aspects of DC physiology^13–16^, the underlying mechanisms remain undefined and the potential interplay between dietary PUFAs, redox homeostasis, and antigen presentation in DCs has not been established. Understanding these mechanisms of immune regulation is fundamental in the context of cancer immunotherapy, especially for aggressive tumor types that remain refractory to this approach. High-grade serous ovarian cancer (HGSOC), for instance, exhibits a highly immunosuppressive tumor microenvironment that inhibits DC function and the induction of protective T cell-mediated anti-tumor responses^17,18^. Whether dietary PUFAs contribute to dysregulated DC activity in this cancer is unknown.

In this study, we investigated the effects of dietary PUFAs on DC function. We found that a diet enriched in n-6 PUFAs profoundly impairs DC activity by promoting ferroptosis through dysregulation of Nrf2-controlled antioxidant responses. Conversely, an n-3 PUFA-enriched diet mitigates ferroptosis and preserves DC function, ultimately improving the efficacy of a DC-based chemoimmunotherapy approach in an aggressive model of HGSOC. Our findings highlight a previously unrecognized link between dietary lipids, ferroptosis, and DC-driven anti-tumor immunity, with implications for cancer immunotherapeutic strategies.

## Results

### Dietary PUFAs shape systemic lipid profiles

**Figure 1.**
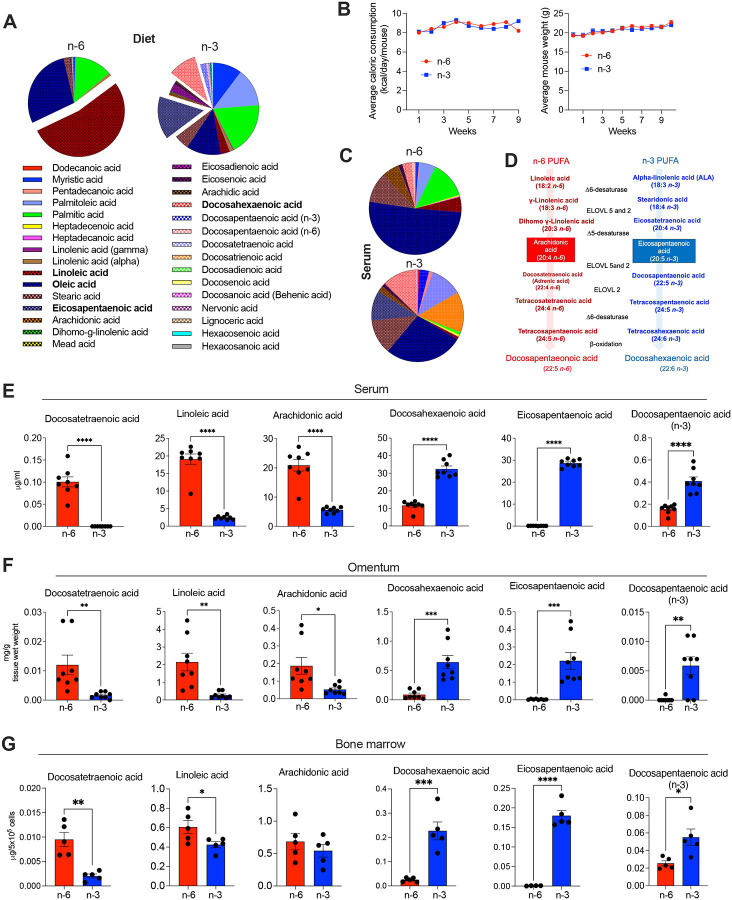
Systemic lipid composition in mice fed diets rich in n-3 or n-6 PUFAs. (A) Lipidomic profiling of the indicated diet pellets. (B) Time-course analysis of cumulative caloric intake (left) and body weight (right) in mice during the dietary intervention period (n = 9 mice per group). (C) Representative pie charts depicting the relative distribution of circulating fatty acids in serum after 9–10 weeks of dietary exposure (n = 8 per group). (D) Schematic of n-6 and n-3 PUFA biosynthetic pathways, highlighting intermediates and enzymes involved in AA and DHA production. (E–G) Quantitative lipidomics of serum (E), omentum (F), and bone marrow cells (G) showing diet-specific enrichment of n-6 and n-3 PUFA species (n = 5–8 per group). Data in (E–G) are presented as mean ± SEM. Statistical significance was determined using unpaired two-tailed Student’s *t*-test: *P < 0.05, **P < 0.01, ***P < 0.001, ****P < 0.0001.

To investigate how dietary lipids shape systemic fatty acid composition, we formulated two isocaloric, semi-synthetic diets differing only in their PUFA content: a corn oil–based diet enriched in n-6 PUFAs and a menhaden oil–based diet enriched in n-3 PUFAs (**Supplemental Table 1**). Gas chromatography–mass spectrometry (GC-MS) analysis of the diets confirmed that the n-6 formulation was predominantly composed of linoleic acid (LA, 18:2n-6, 53%) and lacked EPA and DHA, while the n-3 diet was enriched in eicosapentaenoic acid (EPA, 15.1%) and docosahexaenoic acid (DHA, 10%) and contained minimal LA (Figure 1A; **Supplemental Table 2**). Importantly, mice maintained on these diets for 9–12 weeks showed no differences in caloric intake or body weight (Figure 1B). Thus, we next profiled lipid content across multiple anatomical compartments to determine whether dietary PUFAs were efficiently incorporated into systemic and immune-associated tissues (**Supplemental Table 3**). In the serum, n-6 PUFA–fed mice exhibited significantly higher levels of LA and arachidonic acid (AA), while mice fed a diet rich in n-3 PUFAs showed strong enrichment of EPA, DHA, and docosapentaenoic acid (DPA) (Figure 1C–E). Similar trends were observed in visceral adipose tissue (omentum), where n-6 PUFA–fed mice accumulated more LA and AA, and n-3 PUFA–fed mice incorporated greater levels of EPA and DHA (Figure 1F). Importantly, diet-specific lipid signatures were also evident in total bone marrow cells (Figure 1G). In this hematopoietic compartment, n-6 PUFA–fed mice accumulated docosatetraenoic acid (DTA, 22:4n-6), a long-chain product of AA elongation, while n-3–fed mice incorporated significantly more DPA and DHA (Figure 1G). These data demonstrate that dietary PUFAs are readily incorporated into systemic and cellular lipid pools, establishing a foundation to examine how distinct PUFA profiles influence immune cell biology.

### Dietary PUFAs shape DC function

**Figure 2.**
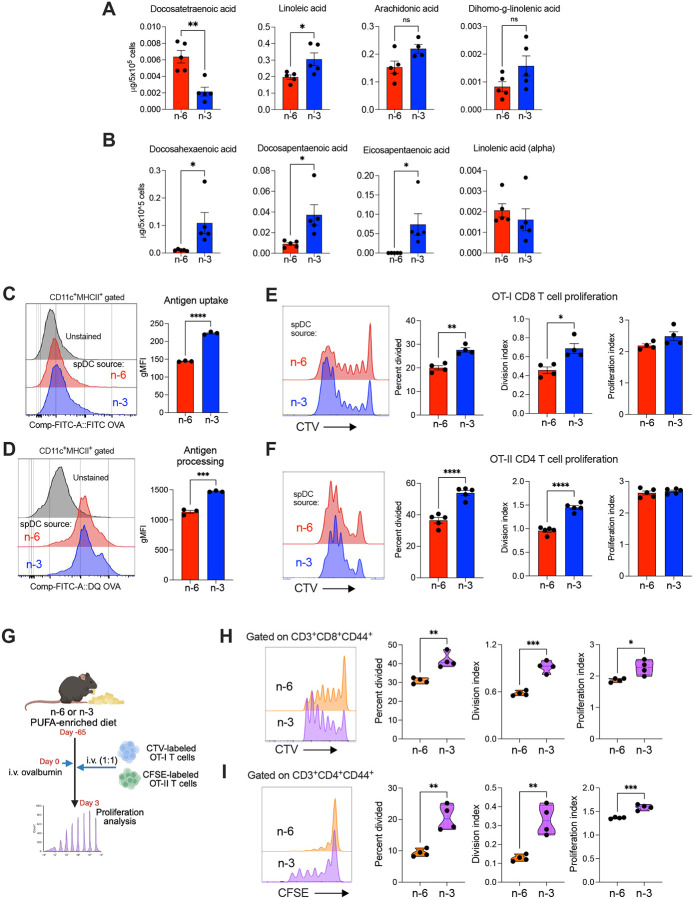
Dietary PUFAs govern DC lipid composition and immunogenic function. Splenic DCs (spDCs) were isolated from mice fed n-6 or n-3 PUFA-enriched diets for 9–10 weeks. (A–B) Quantitative lipidomic analysis of intracellular n-6 and n-3 PUFA species in purified DCs (n = 4–5 per group). (C) Antigen uptake assessed by flow cytometry using fluorescently labeled ovalbumin (OVA); representative histograms and quantification are shown (n = 3 per group). (D) Antigen processing measured by DQ-OVA cleavage (n = 5 per group). (E–F) OVA-pulsed spDCs from the indicated diets were cocultured with OT-I or OT-II T cells for 3 d, and T cell proliferation (CTV dilution) was assessed by flow cytometry; percent divided, division index, and proliferation index are shown (n = 4–5 per group). (G–I) In vivo antigen presentation assays (n = 4 per group). (G) Experimental scheme. (H-I) Representative histograms and violin plots showing CTV and CFSE dilution in proliferating OT-1 (H) and OT-II (I) T cells with quantification of percent divided, division index, and proliferation index. (C-F) Data represent at least two independent experiments with similar results. Data are presented as mean ± SEM. Statistical significance was determined using unpaired two-tailed Student’s *t*-test: *P < 0.05, **P < 0.01, ***P < 0.001, ****P < 0.0001.

To assess whether dietary PUFA intake alters the immune landscape, we performed high-dimensional flow cytometry on splenocytes from mice maintained on n-6– or n-3–enriched diets. n-3 feeding caused a modest but significant increase in splenic B cell frequency, whereas total T cell proportions were relatively higher in n-6–fed mice (**Supplementary Figure 1, A and B**). The abundance of other leukocyte subsets—including monocytes, macrophages, neutrophils, NK cells, total DCs, and DC or T cell subpopulations—was unchanged across diets (**Supplementary Figure 1, A and B**). Given the robust remodeling of systemic and tissue lipidomes (Figure 1), we next asked whether dietary PUFAs influence the DC lipidome, phenotype, and function. Targeted lipidomics of splenic conventional DCs (cDCs) showed diet-specific remodeling where n-6–conditioned DCs accumulated docosatetraenoic acid (22:4n-6; an elongation product of arachidonic acid), whereas n-3–conditioned DCs were enriched in DHA, DPA, and EPA (Figure 2, A and B; **Supplementary Table 3**). Thus, dietary PUFAs are readily incorporated into the DC lipidome, positioning DCs as sensitive responders to nutritional lipid cues. Phenotypic profiling further revealed subset- and diet-dependent changes in costimulatory and antigen-presentation machinery. Splenic cDC1s from mice fed a diet rich in n-3 PUFAs expressed significantly higher levels of CD40, CD80, CD86, and MHC-I than cDC1s from n-6 PUFA-fed mice, both at steady state and following LPS stimulation (**Supplemental Figure 2A**). In cDC2s, expression of CD86 and MHCI was also enhanced in n-3 PUFA-fed mice under both conditions, whereas increased CD40 and CD80 was observed only upon LPS stimulation (**Supplemental Figure 2B**). Splenic DCs from n-3–fed mice exhibited superior antigen handling, with greater uptake of FITC-labeled ovalbumin (OVA) and superior proteolytic processing of DQ-OVA compared with DCs from n-6–fed mice (Figure 2, C and D), indicating increased endocytic and lysosomal activity. We then tested whether these differences translated into improved T cell priming. Notably, full-length OVA-pulsed DCs from n-3–fed mice elicited stronger in vitro proliferation of OT-I (CD8^+^) and OT-II (CD4^+^) T cells than DCs from n-6–fed mice (Figure 2, E and F). To confirm these findings in vivo, diet-conditioned mice were adoptively transferred with CellTrace Violet–labeled OT-I and CFSE-labeled OT-II T cells, followed by intravenous administration of full-length OVA, and T cell proliferation was quantified three days later (Figure 2G). Notably, proliferation of both OT-I and OT-II cells was significantly higher in n-3–fed mice than in their n-6 counterparts (Figure 2, H and I).

We then tested whether the dietary effects could be reproduced by direct lipid exposure. Splenic DCs from chow-fed mice were exposed to defined n-6 fatty acids (linoleic or adrenic; alone or combined) or n-3 fatty acids (DHA or EPA; alone or combined). Under these conditions, the antigen-presenting capacity was minimally changed relative to untreated DCs (**Supplementary Figure 3**), indicating that in vivo conditioning by diet-derived PUFAs—not short-term exposure to isolated fatty acids—is required to elicit the observed differences in DC function. Together, these data reveal that dietary PUFAs have a profound impact on DC antigen uptake, processing, and presentation to T cells.

### DCs programmed by n-3 PUFAs display cytoprotective Nrf2 activation

**Figure 3.**
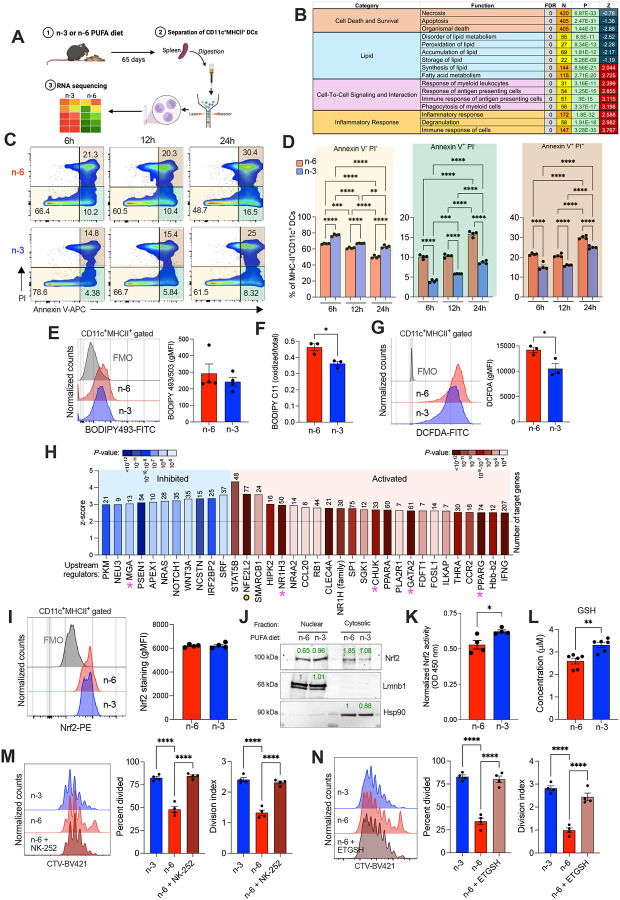
Nrf2 regulates redox homeostasis and preserves DC function according to dietary PUFAs. Mice were maintained on n-6 or n-3 PUFA-enriched diets for 9–10 weeks. Splenic DCs (CD11c^+^MHCII^+^) were sorted and analyzed. (A) Experimental workflow for RNA-seq and mechanistic studies on spDCs from diet-conditioned mice. (B) Ingenuity Pathway Analysis (IPA) of RNA-seq data highlighting enriched and suppressed biological functions in n-3 vs. n-6 spDCs (n = 5 per group). (C–D) Viability of diet-conditioned spDCs assessed over time using Annexin V and PI staining (n = 4 per group). (E) Levels of intracellular neutral lipids measured by BODIPY 493/503 staining (n = 4 per group). (F) Lipid peroxidation assessed by BODIPY 581/591 C11 staining (n = 3 per group). (G) Intracellular ROS levels determined by DCFDA staining (n = 3 per group). (H) IPA-predicted upstream regulators of differentially expressed genes, identifying Nrf2/NFE2L2 (highlighted in yellow circle) as a top upstream regulator in n-3 spDCs. (I) Total NRF2 protein levels in spDCs measured by flow cytometry (n = 4 per group). (J) Representative western blot showing nuclear and cytoplasmic NRF2 localization in spDCs from n-3 and n-6 diet-fed mice. (K) Nrf2 transcriptional activity in nuclear extracts measured by TransAM Nrf2 DNA-binding ELISA (n = 4 per group). (L) Total intracellular glutathione (GSH) levels in the indicated DCs (n = 6 per group). (M) Antigen presentation assay using OVA-pulsed spDCs from n-6 diet-fed mice pretreated with or without the Nrf2 activator (NK-252) and then co-cultured with CellTrace Violet (CTV)-labeled OT-I T cells (n = 4 per group). (N) Similar antigen presentation assay as in (M), with pretreatment using the glutathione donor (ETGSH) (n = 4 per group). CTV dilution was assessed after 72 h of co-culture to evaluate T cell proliferation. Representative histograms, percent divided cells, and division index are shown. (C–G, I–N) Data represent at least two independent experiments. Data in (D–G, I, K–N) are presented as mean ± SEM. Statistical analysis was performed using unpaired two-tailed Student’s t-test (F–G, K–L) or one-way ANOVA with Tukey’s post hoc test (D, M–N): *P < 0.05, **P < 0.01, ***P < 0.001, ****P < 0.0001.

To mechanistically understand how dietary PUFAs dictate DC function, we performed transcriptomic profiling of splenic cDCs sorted from mice fed either the n-6 or n-3 PUFA-enriched diets for 65 days (Figure 3A), a time at which these DCs exhibited diet-specific lipid signatures (Figure 2, A and B). Gene expression analysis revealed 1,246 differentially expressed genes (fold change ≥ 1, *p* < 0.05, FDR ≤ 5%), including 762 transcripts upregulated and 484 transcripts downregulated in n-3 splenic DCs compared with their counterparts from mice fed n-6 PUFAs (**Supplemental Table 4**). Ingenuity Pathway Analysis (IPA) of these genes revealed marked suppression of programs linked to cell death and survival in splenic DCs from n-3 PUFA-fed mice (Figure 3B). Concurrently, lipid peroxidation, lipid storage, and lipid accumulation pathways were inhibited, while biological functions associated with phagocytosis, antigen presentation, inflammatory responses, and myeloid activation were significantly enriched in splenic DCs from mice receiving an n-3 PUFA-enriched diet (Figure 3B). To validate these predictions, we quantified apoptosis, lipid accumulation and peroxidation, and ROS generation in splenic DCs isolated from mice under each diet. Compared with the n-3 PUFA-enriched diet, DCs from n-6 PUFA-fed mice exhibited higher proportions of early and late apoptotic cells at all measured time points (6 h, 12 h, and 24 h post-isolation), confirming increased cell death in the n-6 group (Figure 3, C and D). Notably, while lipid accumulation was unaltered (Figure 3E), n-6 splenic DCs demonstrated a significant increase in lipid peroxidation (Figure 3F) and total ROS levels (Figure 3G), compared with splenic DCs from n-3 PUFA-fed mice.

We next sought to identify the molecular factors driving these transcriptional and phenotypic differences. Upstream regulator analysis indicated that negative transcriptional regulators of DC maturation and immune activation, such as *Irf2bp2*, *Wnt3a*, *Notch1*, and *Psen1*^19–21^, were inhibited in splenic DCs from n-3 PUFA-fed mice (Figure 3H). In contrast, *Nfe2l2* (Nrf2), a transcription factor that coordinates the antioxidant response, emerged as a top activated regulator in DCs programmed by dietary n-3 PUFAs (Figure 3H). Accordingly, splenic DCs from these mice demonstrated robust upregulation of several genes in the glutathione (GSH) biosynthetic and detoxification machinery, including *Gss*, *Gsr*, *Shmt2*, *Mgst1*, *Gstp1*, *Gstm5*, *Gstt2*, and *Gsta4*, as well as classical Nrf2 target genes such as *Nqo1*, *Prdx1*, *Sod3*, *Gpx1*, and *Gpx3* (**Supplemental Figure 4**). Conversely, the oxidative and pro-inflammatory gene *Ptgs2* (encoding Cox2) was downregulated in these DCs compared with their counterparts isolated from mice fed a diet rich in n-6 PUFAs (**Supplemental Figure 4**). This transcriptional profile suggested Nrf2-mediated reinforcement of the GSH antioxidant system as a mechanism by which n-3 PUFAs may protect DCs from redox stress and death, which we sought to functionally test in subsequent experiments.

We found that total Nrf2 protein levels remained unchanged between dietary groups (Figure 3I). However, nuclear fractionation experiments revealed superior Nrf2 translocation in splenic DCs from n-3 PUFA-fed mice (Figure 3J), which was confirmed by enhanced Nrf2 transcriptional activity in an ARE-based DNA binding assay (Figure 3K). Accordingly, the intracellular GSH concentrations were significantly higher in n-3 versus n-6 PUFA-conditioned splenic DCs (Figure 3L). Consistent with these results, unbiased metabolomic analyses demonstrated increased abundance of GSH and GSH-related metabolites, such as L-Cystine, L-Serine, S-Adenosylhomocysteine, and L-Glutamic acid in splenic DCs isolated from mice receiving an n-3 PUFA-rich diet, compared with their counterparts under an n-6 PUFA-based diet (**Supplemental Figure 5**). Of note, splenic DCs isolated from mice fed a diet rich in n-3 PUFAs showed increased expression of genes encoding glutamate (*Slc1a2, Slc1a3*) and glutamine (*Slc38a5, Slc7a8*) transporters, compared with their counterparts isolated from mice under n-6-rich PUFA diets, while expression of genes encoding other cystine, cysteine, glycine, or methionine transporters remained unaltered **(Supplemental Figure 6)**. These results indicate that dietary PUFAs differentially activate Nrf2 signaling in DCs, governing GSH-controlled antioxidant responses. Notably, pharmacologic activation of Nrf2 with NK-252, a compound that disrupts Keap1-mediated degradation of Nrf2^22^ (Figure 3M), or GSH supplementation using glutathione ethyl ester (ETGSH)^23^ (Figure 3N), rescued the defective T cell priming capacity of n-6 splenic DCs to levels comparable with n-3 splenic DCs (Figure 3, M and N). We conclude that Nrf2 is a mechanistic node linking dietary n-3 PUFAs to the preservation of DC redox balance and function, thereby maintaining their optimal capacity to initiate adaptive immunity.

### DCs conditioned by dietary n-6 PUFAs exhibit ferroptosis-driven dysfunction

**Figure 4.**
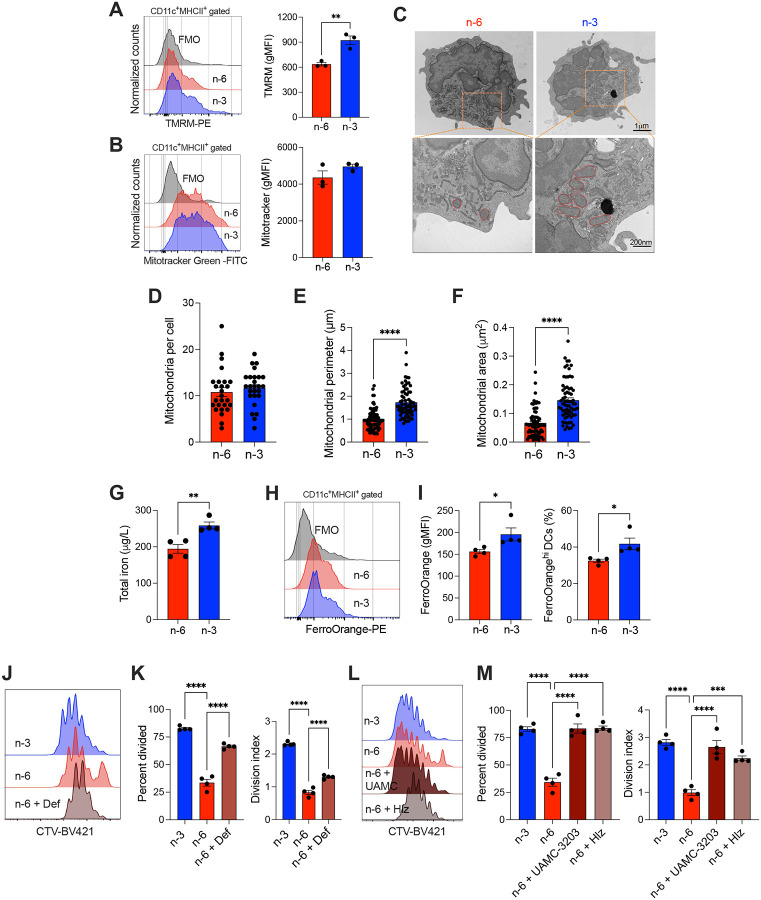
DCs conditioned by an n-6 PUFA–enriched diet exhibit ferroptosis-driven dysfunction. (A–B) Mitochondrial membrane potential and mass measured in spDCs using TMRE and MitoTracker Green staining, respectively; representative histograms and quantification are shown. (C–F) Transmission electron microscopy (TEM) of spDCs isolated from diet-conditioned mice. (C) Representative images of mitochondrial ultrastructure; top row scale bar, 1 μm; bottom row, 200 nm. (D–F) Quantification of mitochondrial number per cell (D), perimeter (E), and area (F). (G–H) Intracellular labile Fe^2+^ was measured in spDCs using FerroOrange staining; representative histograms and geometric mean fluorescence intensity (gMFI) values are shown (n = 4 per group). (I–K) Antigen presentation assays were performed using OVA-pulsed spDCs from n-6 diet-fed mice pretreated with or without the intracellular iron chelator deferiprone, followed by coculture with CellTrace Violet (CTV)-labeled OT-I T cells (n = 4 per group). (L–M) Similar assays as in (I–K), with pretreatment using ferroptosis inhibitor UAMC-3203 or Hydralazine. CTV dilution was measured after 72 hours of co-culture to assess T cell proliferation. Representative histograms, percent of divided cells, and division index are shown. (A–B, G–M) Data represent at least two independent experiments and are presented as mean ± SEM. Statistical significance was determined using unpaired two-tailed Student’s t-test (A–I) or one-way ANOVA followed by Tukey’s post hoc test (D, J–M)): *P < 0.05, **P < 0.01, ***P < 0.001, ****P < 0.0001.

We next sought to understand why splenic DCs isolated from mice receiving a diet rich in n-6 PUFAs show reduced viability and function. GSH is a central regulator of redox homeostasis and a critical suppressor of ferroptosis—a form of regulated cell death driven by lipid peroxidation^24^. As a cofactor for glutathione peroxidase 4 (GPX4), GSH detoxifies lipid hydroperoxides within PUFA-containing phospholipids, preventing membrane damage and cell death^24^. Ferroptosis is fueled by iron metabolism, as ferrous iron (Fe^2+^) reacts with hydrogen peroxide via the Fenton reaction to generate membrane-damaging hydroxyl radicals^25^. Given the elevated oxidative stress and impaired antioxidant capacity observed in splenic DCs programmed by dietary n-6 PUFAs (Figure 3), we next examined whether these cells displayed features of ferroptosis. We evaluated mitochondrial integrity, as ferroptosis is associated with distinct ultrastructural features including mitochondrial shrinkage^26^. Mitochondrial membrane potential, assessed by TMRM fluorescence, was significantly reduced in splenic DCs of n-6 PUFA-fed mice compared with the n-3 PUFA diet (Figure 4A), indicating mitochondrial depolarization. Yet, Mitotracker staining showed no differences in overall mitochondrial mass between groups (Figure 4B). Transmission electron microscopy revealed that while the average number of mitochondria per cell was comparable, splenic DCs in the n-6 PUFA diet displayed reduced mitochondrial perimeter and cross-sectional area relative to the n-3 PUFA diet (Figure 4, C–F), consistent with the compacted, structurally shrunken mitochondria characteristic of ferroptotic cells^26^. We next assessed the cellular iron status. Intriguingly, total and ferrous iron levels were higher in splenic DCs isolated from mice fed a diet rich in n-3 PUFAs, compared with their counterparts on a n-6 PUFA-based diet (Figure 4, G–I). These findings suggest that the enhanced antioxidant capacity of n-3 PUFA–programmed DCs, driven by preserved Nrf2-GSH activity, neutralizes iron-catalyzed oxidative stress. In contrast, DCs conditioned by dietary n-6 PUFAs may be ferroptosis-prone due to heightened ROS reactivity and insufficient detoxification due to defective Nrf2 signaling.

To functionally determine whether ferroptosis underlies DC malfunction under an n-6 PUFA-rich diet, we tested whether chelating iron, quenching ROS, or sequestering lipid peroxidation byproducts could rescue their functionality. Pretreatment with deferiprone (Def), a cell-permeable iron chelator that restricts iron-catalyzed lipid peroxidation^27^, significantly enhanced the antigen-presenting capacity of splenic DCs isolated from mice fed an n-6 PUFA-rich diet (Figure 4, J and K). Treatment with UAMC-3203, a radical-trapping antioxidant that stabilizes lipid radicals in membranes and prevents ferroptosis^28^, also restored antigen presentation by DCs conditioned by dietary n-6 PUFAs (Figure 4, L and M). Furthermore, hydralazine (Hlz)—a hydrazine derivative that quenches lipid peroxidation byproducts^29,30^—also rescued their T cell priming capacity (Figure 4, L and M). Collectively, these findings indicate that DCs from n-6 PUFA–fed mice exhibit mitochondrial defects, redox imbalance, and ferroptosis-associated dysfunction driven by a defective Nrf2-GSH axis.

### Dietary PUFAs dictate the efficacy of DC-based chemoimmunotherapy in ovarian cancer

Splenic DCs from mice fed an n-3 PUFA–enriched diet exhibited elevated Nrf2 activity, increased GSH pools, and enhanced antigen-presenting capacity (Figure 3), features consistent with a cytoprotective phenotype capable of resisting oxidative stress. We hypothesized that this redox-resilient state may enable n-3 PUFA–conditioned DCs to maintain immunogenic function in hostile environments. Ovarian cancer provides a stringent setting to test this hypothesis, as it imposes profound oxidative stress on immune cells and drives DC dysfunction to suppress protective anti-tumor immunity^30–32^. Importantly, while adoptive DC-based immunotherapies have demonstrated only modest benefit in ovarian cancer patients^33,34^, these clinical limitations may stem from the susceptibility of DCs to redox-driven dysfunction within the tumor microenvironment. Based on our mechanistic findings, we posited that dietary PUFA conditioning could influence the immunotherapeutic efficacy of adoptively transferred DCs. To test this, we evaluated DCs from mice fed either an n-3 or n-6 PUFA–enriched diet in a murine model of metastatic high-grade serous ovarian carcinoma (HGSOC), the most common and aggressive form of ovarian cancer^35^.

To validate the therapeutic relevance of dietary programming, we first evaluated whether bone marrow-derived DCs (BMDCs)—commonly used in preclinical immunotherapy protocols—retain the same immunometabolic imprinting observed in splenic DCs. Transcriptomic analyses revealed that BMDCs generated from mice under an n-3 PUFA-rich diet exhibited suppression of gene programs associated with cell death, lipid accumulation, and apoptosis of myeloid cells, while pathways linked to phagocyte activation, leukocyte interactions, antigen presentation, and cellular differentiation were significantly enriched (**Supplemental Figure 7A**). These findings mirror the transcriptional reprogramming observed in splenic DCs (Figures 3 and 4), demonstrating that dietary PUFA–induced immunometabolic signatures are conserved across DC compartments. Consistent with their transcriptional profile, BMDCs generated from mice fed a n-3 PUFA-rich diet also exhibited significantly higher intracellular GSH levels than BMDCs from n-6 PUFA–fed mice, both at baseline and following LPS stimulation (**Supplemental Figure 7, B and C**). Moreover, n-3 PUFA BMDCs elicited significantly stronger proliferation of OT-II CD4^+^ and OT-I CD8^+^ T cells in vitro (**Supplemental Figure 7, D–G**), confirming that the enhanced immunogenic capacity observed in splenic DCs is maintained in BMDC preparations.

**Figure 5.**
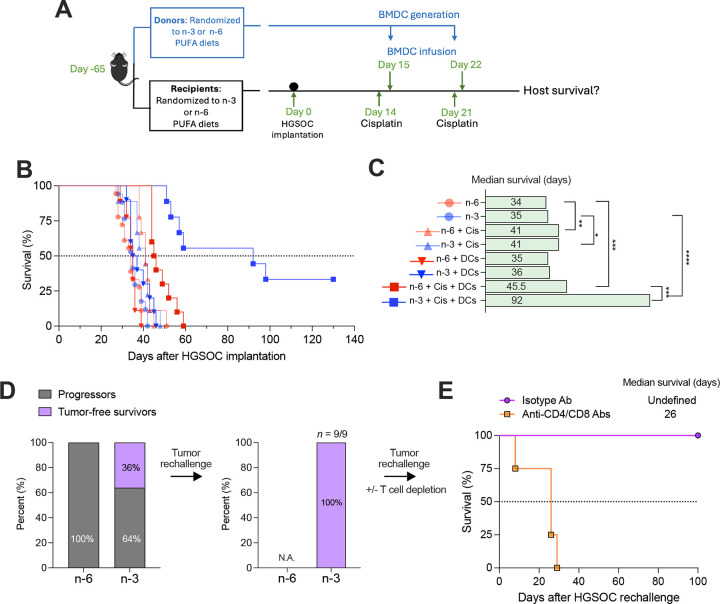
DCs from mice fed a diet rich in n-3 PUFAs elicit durable anti-tumor immunity. (A) Schematic of in vivo experimental workflow: mice maintained on n-3 or n-6 PUFA diets were challenged i.p. with PPNM HGSOC cells, then treated with cisplatin followed by I.P. delivery of matched BMDCs. Two weeks after tumor implantation, recipient mice (n = 9–17 per group) were treated as indicated. BMDCs were obtained from mice under the corresponding diets for the same length and infused I.P. at 3 × 10^6^ cells/mouse each cycle. (B–C) Kaplan–Meier survival analysis of tumor-bearing mice (n = 9–17 per group) treated with cisplatin and/or BMDCs from the indicated diets. (D) Long-term survivors (n = 9 of 25 pooled from two independent experiments) were rechallenged I.P. with PPNM cells at day 120. Mice were monitored for an additional 120 days; the proportion of tumor rejectors is shown on the right. (E) Tumor rejectors from (D) were randomized to receive either isotype control (n = 5) or CD4 plus CD8 T cell–depleting antibodies (n = 4) every 3 days for 2 weeks, followed by a third tumor rechallenge. Kaplan–Meier survival is shown. *P < 0.05, **P < 0.01, ***P < 0.001, ****P < 0.0001.

We next tested whether these immunometabolic differences impacted the therapeutic efficacy of DC-based immunotherapy in a mouse model of HGSOC. To this end, female mice fed n-3 or n-6 PUFA–enriched diets were orthotopically implanted with *Trp53*^R172H^*Pten*^−/−^*Nf1*^−/−^*Myc*^OE^ (PPNM) tumors that model metastatic high-grade serous tubo-ovarian cancer^36^ (Figure 5A). Two weeks later, mice received intraperitoneal cisplatin chemotherapy followed by infusion of BMDCs derived from donor mice on the corresponding diet (Figure 5A). BMDCs were delivered intraperitoneally to ensure direct access to tumor antigens released after chemotherapy within this compartment. HGSOC progression and overall host survival were comparable in untreated mice receiving either diet (Figure 5, B and C), denoting that diet alone is insufficient to alter HGSOC progression in this aggressive model. Negligible therapeutic effects were observed in groups receiving either cisplatin or adoptive infusion with BMDCs generated from donor mice under the corresponding diets (Figure 5, B and C). These data confirm that PPNM tumors are highly resistant to cisplatin and imply that DC transfer alone is insufficient to induce anti-tumor effects in either diet. Combination therapy with cisplatin and BMDCs generated from donors receiving n-6 PUFA-rich diets also failed to control HGSOC in mice under the same diet (Figure 5, B and C). Strikingly, however, when mice were fed a diet rich in n-3 PUFAs, this DC-based chemoimmunotherapy elicited marked anti-cancer effects characterized by a significant increase in median survival (92 days), and approximately 36% of treated mice demonstrating complete tumor elimination (Figure 5, B and C). Notably, all long-term survivors resisted rechallenge with HGSOC cells (Figure 5D), confirming the generation of protective memory responses. Of note, this protective effect was abolished upon antibody-mediated T cell depletion, demonstrating the role of adaptive immunity in long-term tumor control (Figure 5E).

Together, these findings demonstrate that dietary PUFAs shape DC phenotype and function, influencing the in vivo efficacy of DC-based chemoimmunotherapy. While n-6 PUFA–conditioned DCs fail to control tumor growth, DCs programmed by dietary n-3 PUFAs synergize with cisplatin to elicit durable, T cell–dependent anti-tumor immunity. These results underscore the potential of nutritional interventions to enhance the therapeutic impact of cellular immunotherapies in treatment-resistant malignancies and those with high recurrence rates such as ovarian cancer.

## Discussion

Our study uncovers a diet-sensitive metabolic checkpoint in DCs that governs their survival and immunogenic function through the regulation of ferroptosis. Specifically, we demonstrate that dietary PUFAs reprogram the systemic lipid composition and, in doing so, differentially regulate the redox balance of DCs via the Nrf2–glutathione axis. These findings bridge nutrition, DC function, and immunotherapeutic tumor control.

PUFAs are essential lipid components of cell membranes and metabolic signaling networks, but their role in regulating immune cell viability and homeostasis has remained largely unexplored. We found that an n-6 PUFA–enriched diet increases the abundance of pro-ferroptotic lipid species, including AA and adrenic acid, in DCs. These substrates are prone to peroxidation^37^, promoting oxidative damage and mitochondrial anomalies. In contrast, DHA and EPA are preferentially incorporated into DCs under n-3 PUFA-enriched diets, mediating resistance to ferroptosis through enhanced Nrf2 activation and GSH preservation.

A central mechanistic insight from our work is that Nrf2 functions as a dietary sensor and redox modulator in DCs. In n-3 PUFA-fed mice, Nrf2 activation promoted the transcription of antioxidant enzymes, maintained intracellular GSH levels, and suppressed lipid peroxidation. This protects DCs from ferroptosis and safeguards their ability to activate naïve CD4+ and CD8+ T cells. Notably, pharmacologic activation of Nrf2, exogenous GSH supplementation, or ferroptosis inhibition rescued the APC function of DCs from n-6 PUFA-fed mice, emphasizing the role of Nrf2 as gatekeeper of this axis.

Importantly, the impact of dietary PUFAs extended beyond DC homeostasis to therapeutic anti-tumor immunity. We found that only DCs programmed by an n-3 PUFA–enriched diet, when adoptively transferred following cisplatin chemotherapy, elicited robust control of HGSOC and durable adaptive immunity that conferred protection against tumor rechallenge. This synergy highlights the therapeutic relevance of preserving DC redox integrity to enhance their function in cancer therapeutic vaccines or adoptive immunotherapy. Our findings also raise the major possibility of using nutritional adjuvants that bolster immunotherapy through DC ferroptosis suppression.

Our study expands the scope of ferroptosis research into the realm of professional antigen-presenting cells. The identification of Nrf2 as a master regulator in this context provides a mechanistic entry point for modulating DC viability and function in diseases ranging from cancer to infection and autoimmunity. Our findings suggest that dietary interventions could be tailored to shape immune outcomes by tuning susceptibility to ferroptosis. Given the Western diet’s bias toward n-6 PUFAs, these discoveries provide a mechanistic rationale to reconsider nutritional guidelines in immunologically vulnerable populations, such as cancer patients.

## Methods

### Mouse studies

Female C57BL/6J, OT-I (C57BL/6-Tg(TcraTcrb)1100Mjb/J), and OT-II (B6.Cg-Tg(TcraTcrb)425Cbn/J) transgenic mice were purchased from The Jackson Laboratory (Bar Harbor, ME) and housed in pathogen-free microisolator cages at the Weill Cornell Medicine Animal Facility. Mice were maintained under a 12-hour light/dark cycle with ad libitum access to water and assigned diets, and all animal experiments were approved by the Institutional Animal Care and Use Committee (IACUC) at Weill Cornell Medicine (protocol number 2011–0098). All studies were conducted using female mice. To normalize microbiota composition across groups and reduce cage effects, 5- to 6-week-old C57BL/6J mice underwent a 2-week bedding exchange protocol, where bedding was swapped between cages within the cohort two times per week. Following this normalization period, mice were randomized into dietary intervention groups. Mice were fed either an n-6 polyunsaturated fatty acid (PUFA)-enriched diet (Research Diets, D18062204; 16% calories from corn oil) or an n-3 PUFA-enriched diet (Research Diets, D12041401; 16% calories from menhaden fish oil) for 8 to 12 weeks. Diets were matched for macronutrient composition to ensure that differences between groups were attributable to fatty acid composition. Mice were monitored for weight and general health throughout the intervention period. At the end of the dietary intervention, mice were sacrificed, and tissues were collected for downstream metabolic, immunologic, and molecular analyses.

### Primary Cell Isolation and Analysis

Spleens were collected from mice fed either the n-6 or n-3 diet, homogenized, and enzymatically digested with collagenase D (2.5 mg/ml, Roche) and DNase I (0.05 mg/ml, Sigma-Aldrich) at 37°C for 30 minutes. Following digestion, single-cell suspensions were prepared by passing the cells through 70 μm cell strainers, and red blood cells (RBCs) were lysed using ammonium-chloride-potassium (ACK) buffer (Life Technologies). Total splenic DCs were isolated using a magnetic Pan Dendritic Cell Isolation Kit (Miltenyi Biotec, 130-100-875) following the manufacturer’s instructions. Murine BMDCs were generated from bone marrow precursor cells isolated from the tibiae and femurs of mice. Bone marrow cells were flushed, and RBCs were lysed using ACK buffer. BM cells were then plated on bacteriologic plates at 3 × 10^6^/plate in 10 mL of complete RPMI media (RPMI + L-glutamine + 10% FBS + HEPES + sodium pyruvate + nonessential amino acids + β mercaptoethanol + penicillin/streptomycin) containing 20 ng/mL of recombinant granulocyte–macrophage colony-stimulating factor (GM-CSF; PeproTech; cat.#315–03). Three days later, an equal volume of the media described above was added to the culture, and nonadherent cells were harvested 4 days thereafter (day 7). BMDCs were further enriched with Pan Dendritic Cell Isolation Kit (Miltenyi Biotec, 130-100-875) and used directly for downstream assays.

For T cell isolation, OT-I and OT-II transgenic mice were used to obtain CD8^+^ and CD4^+^ T cells, respectively. Spleens and lymph nodes (submandibular, axillary, and inguinal) were harvested, mechanically dissociated, and processed into single-cell suspensions using a syringe plunger and passed through 70 μm strainers. RBCs were lysed using ACK buffer (Life Technologies). Naïve CD8^+^ and CD4^+^ T cells were isolated by magnetic negative selection using the Naïve CD8^+^ T Cell Isolation Kit, Mouse (Miltenyi Biotec, 130-096-543) and the Naïve CD4^+^ T Cell Isolation Kit, Mouse (Miltenyi Biotec, 130-104-453), respectively.

### Antigen Presentation and T Cell Proliferation Assays

Freshly isolated CD11c^+^ splenic DCs were obtained by magnetic purification (Pan DC Isolation Kit, Miltenyi Biotec) and pulsed with full-length ovalbumin (EndoFit^™^ OVA protein 0.1 mg/ml, InvivoGen Cat# vac-pova) at 37°C for 6 hours. For experiments involving inhibitors, DCs were preincubated for 1 hour with the following compounds prior to OVA pulsing: Deferiprone (100 μM; Sigma-Aldrich, Cat# 379409), an intracellular iron chelator; Glutathione ethyl ester (Et-GSH) (0.5 mM; Cayman Chemical, Cat# 14953), a cell-permeable glutathione derivative; UAMC-3203 (100 nM; Cayman Chemical, Cat# 26525), a ferroptosis inhibitor; Hydralazine (100 μg/ml; Sigma-Aldrich, Cat# H1753–5G), a hydrazine derivative that sequesters reactive lipid peroxidation byproducts; NK-252 (10 μM; MedChemExpress, Cat# HY-19734), a specific activator of the transcription factor Nrf2. CD8^+^ and CD4^+^ T cells were isolated from lymphoid tissues (spleen, submandibular, axillary, and inguinal lymph nodes) of OT-I and OT-II transgenic mice, respectively, using negative selection kits (Naïve CD8^+^ T Cell Isolation Kit, Miltenyi Biotec, Cat# 130-096-543; Naïve CD4^+^ T Cell Isolation Kit, Miltenyi Biotec, Cat# 130-104-453). Isolated T cells were labeled with CellTrace Violet (2.5 μM, Thermo Fisher Scientific Cat#C34571) by incubating cells at 37°C for 15 minutes in the dark. Staining was quenched by adding 2 volumes of RPMI-1640 medium supplemented with 10% FBS, followed by a 5-minute incubation at 37°C in the dark. Labeled cells were washed with complete RPMI-1640 medium containing 10% FBS. OVA-pulsed DCs were co-cultured with CellTrace Violet-labeled naïve CD8^+^ or CD4^+^ T cells at a 1:5 DC:T cell ratio. Co-cultures were maintained at 37°C for 72 hours. T cell proliferation was assessed by flow cytometry, measuring CellTrace Violet dilution peaks using FlowJo software. To quantitatively evaluate proliferation, the main peak of the RPMI-1640 control was designated as the parent (zeroth generation) population. Fluorescence ratios between successive peaks were fixed, and a consistent coefficient of variation (CV) was applied across all generations. Percent divided reflects the precursor frequency, i.e., the proportion of cells that underwent division. The division index was defined as the average number of divisions per cell in the starting population. The proliferation index was calculated as the total number of divisions divided by the number of dividing cells.

### In vivo Antigen Presentation Assays

Mice maintained on either an n-6– or n-3–enriched diet were injected intravenously with 125 μg of full-length ovalbumin (EndoFit^™^ OVA protein, 0.1 mg/ml; InvivoGen, Cat# vac-pova). Naïve CD8^+^ and CD4^+^ T cells were isolated from spleens and lymph nodes (submandibular, axillary, and inguinal) of OT-I and OT-II transgenic mice, respectively, using magnetic negative selection kits (Miltenyi Biotec; CD8^+^, Cat# 130-096-543; CD4^+^, Cat# 130-104-453). Purified CD8^+^ and CD4^+^ T cells were labeled with CellTrace^™^ Violet (CTV; 5 μM; Thermo Fisher Scientific, Cat# C34571) or CFSE (5 μM; Thermo Fisher Scientific, Cat# C34570), respectively, by incubation at 37 °C for 20 min in the dark. Labeling was quenched with RPMI-1640 medium containing 10% FBS for 5 min at 37 °C, followed by washing. Equal numbers of CD8^+^ and CD4^+^ T cells were mixed at a 1:1 ratio, and a total of 6 × 10⁶ labeled T cells were injected intravenously into OVA-injected recipient mice. After 3 days, mice were euthanized, and spleens and lymph nodes were harvested to prepare single-cell suspensions. Following RBC lysis, cells were washed and incubated with Fc receptor blocker (TruStain FcX^™^, anti-mouse CD16/32; BioLegend, clone 93, Cat# 101319) for 10 min at 4 °C, then stained with a panel of surface markers for 30 min at 4 °C: CD45 (BioLegend, clone 30-F11, Cat# 103128), CD3 (BD Biosciences, clone 145–2C11, Cat# 612771), CD4 (BioLegend, clone GK1.5, Cat# 100455), CD8 (BioLegend, clone 53–6.7, Cat# 100747), CD44 (BioLegend, clone IM7, Cat# 103012), CD62L (BioLegend, clone MEL-14, Cat# 104408), CD11c (BioLegend, clone N418, Cat# 117328), Ly-6G (Tonbo Biosciences, clone 1A8, Cat# 65–1276-U025), NK1.1 (BioLegend, clone PK136, Cat# 108728), F4/80 (BioLegend, clone BM8, Cat# 123128), and B220 (BioLegend, clone RA3–6B2, Cat# 103235). After washing, cells were stained with LIVE/DEAD^™^ Fixable Near-IR Dead Cell Stain (Invitrogen^™^, Cat# L10119) for 20 min at 4 °C. Flow cytometric analysis was performed on a BD LSRFortessa instrument, and T cell proliferation was quantified based on CTV or CFSE dilution using FlowJo software (BD Biosciences).

### Ex vivo fatty acid treatment

Splenic dendritic cells (DCs) were isolated from chow diet–fed C57BL/6 mice and cultured overnight with specific n-6 or n-3 polyunsaturated fatty acids (PUFAs). Fatty acids were complexed with 1% fatty acid–free bovine serum albumin (BSA; Sigma-Aldrich, Cat# A8806) and added to cultures at a final concentration of 50 μM. The following fatty acids were used: linoleic acid (LA; Sigma-Aldrich, Cat# L1012), adrenic acid (AdA; Sigma-Aldrich, Cat# D3659), eicosapentaenoic acid (EPA; MedChem Express, Cat# HY-B0660), and docosahexaenoic acid (DHA; MedChem Express, Cat# HY-B2167). After 18 hours of incubation, DCs were pulsed with ovalbumin (OVA) for an additional 6 hours to allow antigen uptake and processing. Cells were then thoroughly washed to remove residual fatty acids, and purified CD8^+^ T cells from OT-I transgenic mice were co-cultured with the treated DCs for assessment of antigen presentation and T cell proliferation, as described above.

### RNA Sequencing and Transcriptomic Analyses

Splenic DCs and BMDCs were isolated from mice maintained on n-6 or n-3 diets. DCs were magnetically purified and then further sorted for CD45^+^CD11c^+^MHCII^+^ populations using a FACSAria instrument (BD Biosciences). Total RNA was extracted using the RNeasy Plus Micro Kit (Qiagen), and RNA quality was assessed using the Agilent Bioanalyzer 2100. Library preparation and sequencing were performed at the Weill Cornell Genomics Resources Core Facility. For splenic DCs, libraries were prepared using the SMART-Seq v4 Ultra Low Input RNA Kit followed by the Nextera XT DNA Sample Preparation Kit. For BMDCs, libraries were prepared using the NEB Ultra II Directional RNA Library Prep Kit with the Poly(A) isolation module. Sequencing was conducted using Illumina HiSeq2500 with high-throughput (HT) DNA sequencing. For quality control and read processing, Quality control (QC) was performed using MultiQC^38^ and adapter sequences were removed from raw reads using cutadapt^39^. RNA sequencing reads were aligned using the bowtie2 algorithm^40^ via RSEM v1.3.3^41^ to estimate read counts and RPKM (Reads Per Kilobase of transcript, per Million mapped reads) values, utilizing the Ensembl GRCm38 transcriptome. For pathway analysis, raw read counts were used to determine differential gene expression between experimental groups using DESeq2^42^. Gene expression differences were considered statistically significant if *P* < 0.05. Gene set enrichment analysis (GSEA) was performed using QIAGEN’s Ingenuity^®^ Pathway Analysis (IPA^®^) software (QIAGEN Redwood City, www.qiagen.com/ingenuity). Pathway analyses were conducted using “Canonical Pathways,” “Diseases & Functions,” and “Upstream Regulators” modules. Pathways predicted to be activated based on a positive Z-score for predicted activation state were reported. All bioinformatic analyses were conducted at the Bioinformatics Facility of The Wistar Institute. Sequencing data have been deposited under NCBI GEO accession number GSE292400.

### Phagocytosis and Antigen Processing Assays

Freshly isolated splenic DCs from mice fed either the n-6 rich diet or n-3 rich diet were incubated at 37°C for 3 hours with fluorescein-conjugated ovalbumin (5 μg/ml; Thermo Fisher Scientific, Cat# O23020) in a CO_2_ incubator to assess phagocytic capacity. For the antigen processing assay, DCs were incubated with DQ-ovalbumin (DQ-OVA, 50 μg/ml; Thermo Fisher Scientific, Cat# D12053) at either 0°C or 37°C for 3 hours. Following incubation, cells were washed twice with cold phosphate-buffered saline (PBS) and incubated with TruStain FcX^™^ (anti-mouse CD16/32, BioLegend, clone 93, Cat# 101319) for 10 minutes at 4°C to block Fc receptors. Cells were subsequently stained with: CD45 (BioLegend, clone 30-F11, Cat# 103128), CD11c (BioLegend, clone N418, Cat# 117308), I-A/I-E (MHC class II) (BioLegend, clone M5/114.15.2, Cat# 107626). Phagocytic uptake and antigen processing were quantified by flow cytometry, using fluorescence intensity within the CD45^+^CD11c^+^MHC-II^+^ population. To distinguish active uptake and processing from passive surface binding, data from cells incubated at 37°C were compared to cells incubated at 0°C. Results were further compared between DCs isolated from n-6 and n-3 diet-fed mice to assess diet-dependent differences in antigen uptake and processing efficiency.

### Intracellular ROS Assays

Intracellular reactive oxygen species (ROS) levels were measured using 2′,7′-dichlorofluorescein diacetate (DCFDA) (Thermo Fisher Scientific, Cat# C6827). Freshly isolated splenic DCs from mice fed either the n-6 rich diet or n-3 rich diet were resuspended in complete RPMI medium at 2.5 × 10^5^ cells per well. Cells were incubated with DCFDA (10 μM) for 30 minutes at 37°C in the dark. Immediately following incubation, fluorescence intensity was quantified by flow cytometry, measuring DCF fluorescence as a readout of intracellular ROS levels. Data were analyzed using FlowJo software, and comparisons were made between dietary groups to assess diet-induced differences in ROS generation.

### Lipidomic Analyses

Lipidomics were performed on splenic DCs and total bone marrow cells isolated from mice fed either an n-6 PUFA rich diet or n-3 rich diet for 8 weeks. Splenic DCs were magnetically purified and then further sorted for CD45^+^CD11c^+^MHCII^+^ populations using a FACSAria instrument (BD Biosciences). Total bone marrow cells were collected after RBCs lysis using ACK buffer. Cell pellets (50,000 cells) were washed with PBS, and frozen at −80°C until further processing. Total fatty acid profiling was conducted to quantify individual fatty acids and lipid species using liquid chromatography-mass spectrometry (LC-MS). All lipidomic analyses were performed at the Lipidomics Core Facility of Wayne State University School of Medicine. Data were analyzed to compare lipid composition across dietary groups, with a particular focus on the incorporation of n-6 and n-3 PUFAs into cellular lipids.

### Targeted metabolomics

Freshly isolated splenic DCs from mice fed either the n-6 rich diet or n-3 rich diet were magnetically purified and then further sorted for CD45^+^CD11c^+^MHCII^+^ populations using a FACSAria instrument (BD Biosciences). Cells (1.4×10^6^) were pooled by group (n=7–8 mice/group) and frozen at −80°C until further processing. The sample was extracted using pre-chilled 80% methanol (−80 °C). The extract was dried with a Speedvac, and redissolved in HPLC grade water before it was applied to the hydrophilic interaction chromatography LC-MS. Metabolites were measured on a Q Exactive Orbitrap mass spectrometer (Thermo Scientific), which was coupled to a Vanquish UPLC system (Thermo Scientific) via an Ion Max ion source with a HESI II probe (Thermo Scientific). A Sequant ZIC-pHILIC column (2.1 mm i.d. × 150 mm, particle size of 5 μm, Millipore Sigma) was used for separation of metabolites. A 2.1 × 20 mm guard column with the same packing material was used for protection of the analytical column. Flow rate was set at 150 μL/min. Buffers consisted of 100% acetonitrile for mobile phase A, and 0.1% NH4OH/20 mM CH3COONH4 in water for mobile phase B. The chromatographic gradient ran from 85% to 30% A in 20 min followed by a wash with 30% A and re-equilibration at 85% A. The Q Exactive was operated in full scan, polarity-switching mode with the following parameters: the spray voltage 3.0 kV, the heated capillary temperature 300 °C, the HESI probe temperature 350 °C, the sheath gas flow 40 units, the auxiliary gas flow 15 units. MS data acquisition was performed in the m/z range of 70–1,000, with 70,000 resolution (at 200 m/z). The AGC target was 1e6 and the maximum injection time was 250 ms. The MS data was processed using XCalibur 4.1 (Thermo Scientific) to obtain the metabolite signal intensities. Identification required exact mass (within 5ppm) and standard retention times.

### Total Iron Quantification

Splenic DCs were isolated from mice fed either an n-6-rich or an n-3-rich diet for 9–10 weeks. DCs (1×10^6^) were then pelleted and stored in −80°C until further processing. Samples were digested using 50% nitric acid and then heated at 70°C for 2 hours in a heat block. The digested samples were then cooled to room temperature and centrifuged for 5 minutes at 6000 xg. The supernatant was collected and diluted in 0.2% nitric acid for measurement via a graphite furnace atomic absorption spectrometer (PerkinElmer model 900z). Iron was measured in the digestion buffer to assess iron contamination, and sample iron levels are quantified using a standard curve of known iron concentrations.

### Cell Viability Assays

Splenic DCs and BMDCs were isolated from mice fed either the n-6 or n-3 rich diet for 8 weeks using magnetic purification with the Pan DC Isolation Kit (Miltenyi Biotec, Cat# 130-100-875). Purified DCs were cultured in complete RPMI medium at 37°C in a 5% CO₂ incubator for time-course viability assessments. Cells were blocked using TruStain FcX^™^ (anti-mouse CD16/32) (BioLegend, Clone 93, Cat# 101319) followed by surface-staining with: CD11c (BD Biosciences, Clone N418, Cat# 744180) and I-A/I-E (MHC Class II) (BioLegend, Clone M5/114.15.2, Cat# 107620). After staining, cells were washed twice with PBS and assessed for viability using the APC Annexin V Apoptosis Detection Kit with Propidium Iodide (PI) (BioLegend, Cat# 640932), following the manufacturer’s instructions. Cell viability was quantified by flow cytometry. Data were analyzed using FlowJo software, and comparisons were made across dietary groups.

### Glutathione Quantification

Splenic DCs were isolated from mice fed either an n-6 or n-3 rich diet using magnetic purification with the Pan DC Isolation Kit (Miltenyi Biotec, Cat# 130-100-875). Isolated cells (1 × 10⁶ cells per condition) were lysed in PBS containing 0.5% NP-40, and lysates were deproteinized using the Deproteinizing Sample Preparation Kit – TCA (Abcam, Cat# ab204708). Deproteinized lysates were used to quantify total glutathione levels using the Glutathione Assay Kit (Abcam, Cat# ab138881) according to the manufacturer’s instructions. Fluorescence was monitored at Ex/Em = 490/520nm using SpectraMax iD3 instrument (Molecular Devices).

### Flow Cytometry

Single-cell suspensions were washed in ice-cold FACS buffer (phosphate-buffered saline [PBS] supplemented with 2% fetal bovine serum [FBS] and 2 mM EDTA) and incubated with TruStain FcX^™^ (anti-mouse CD16/32; BioLegend, Clone 93, Cat# 101319) at 4°C for 10 minutes to block Fcγ receptors and minimize nonspecific binding. Surface staining was performed at 4°C for 30 minutes in the dark using the following fluorochrome-conjugated antibodies (BioLegend unless otherwise noted): CD45 (Clone 30F11; BD Biosciences, Cat# 562420), CD3 (Clone 17A2; Cat# 100216), CD19 (Clone ID3; BD Biosciences, Cat# 612781), CD11b (Clone M1/70; Cat# 101257), F4/80 (Clone BM8; Cat# 123110), CD11c (Clone N418; BD Biosciences, Cat# 744180), I-A/I-E (MHC class II) (Clone M5/114; Cat# 107620), Ly6C (Clone HK1.4; Cat# 128041), CD4 (Clone GK1.5; Cat# 100414), CD8α (Clone 53–6.7; Cat# 100706), Ly6G (Clone 1A8; Tonbo Biosciences, Cat# 20–1276-U100), NK1.1 (Clone PK136; Cat# 108748), CD86 (Clone GL-1; Cat# 105030), CD80 (Clone 16–10A1; Cat# 104734), CD40 (Clone 3/23; Cat# 124612), and CD71 (Clone C2; BD Biosciences, Cat# 740223). Following staining, cells were washed with PBS, and dead cells were excluded by staining with DAPI. For intracellular staining, Cells were first stained for surface markers as described above, followed by viability assessment using the Live/Dead Fixable Blue Dead Cell Stain (Thermo Fisher Scientific, Cat# L34962) according to the manufacturer’s protocol. Cells were then fixed and permeabilized using the FOXP3/Transcription Factor Staining Buffer Set (eBioscience, Cat# 00-5523-00) per the manufacturer’s instructions. For intracellular detection of NRF2, cells were incubated overnight at 4°C with anti-NRF2 rabbit monoclonal antibody (Thermo Fisher Scientific, Cat# 12721, 1:100 dilution). Following primary antibody incubation, cells were washed and stained with PE-conjugated goat anti-rabbit IgG (H+L) secondary antibody (Thermo Fisher Scientific, Cat# P-2771MP, 1:200 dilution) for 30 minutes at room temperature in the dark. Cells were washed and resuspended in FACS buffer prior to analysis by flow cytometry.

For neutral lipid quantification, splenic DCs (2 × 10^5^ cells) were incubated with BODIPY 493/503 (Thermo Fisher, Cat# D3922, 0.5 mg/mL in PBS) for 15 min at room temperature in the dark. For oxidized lipid measurement, DCs were incubated with 4 μM BODIPY 581/591 C11 (Lipid Peroxidation Sensor) (Thermo Fisher, Cat# D3861) for 30 min at room temperature in PBS. To assess mitochondrial mass, DCs (1 × 10^5^ cells) were first stained for surface markers, followed by incubation with 100 nM MitoTracker Green (Thermo Fisher Scientific, Cat# M46750) for 30 min at 37°C. For mitochondrial membrane potential, DCs were stained with 10 nM TMRM (Thermo Fisher Scientific, Cat# M20036) for 30 min at 37°C, followed by DAPI staining for dead cell exclusion. For reducing iron quantification, DCs were washed with serum-free media and incubated with 1 μmol/L FerroOrange (Dojindo Laboratories, Cat# F374) for 30 min at 37°C in a CO_2_ incubator. Flow cytometry was performed using a Fortessa-X20 cytometer (BD Biosciences), and data were analyzed using FlowJo v.10 (TreeStar).

### Transmission Electron Microscopy (TEM)

CD45^+^CD11c^+^MHC-II^+^ splenic DCs were sorted from mice fed either an n-6 or n-3 rich diet. Following isolation, cells were washed twice with PBS, and cell pellets were collected for electron microscopy processing. Cells were fixed, embedded, sectioned, and stained according to standard protocols^30^. All TEM imaging and sample preparation were performed at the Electron Microscopy and Histology Core Facility of Weill Cornell Medical College. Images were acquired and analyzed to assess mitochondrial ultrastructural features.

### Western Blotting

Splenic DCs isolated from mice fed either n-6 or n-3 PUFA rich diet were subjected to nuclear and cytoplasmic fractionation using the NE-PER Nuclear and Cytoplasmic Extraction Kit (Thermo Fisher Scientific, Cat# 78833) following the manufacturer’s instructions. Protein concentrations were determined using a BCA Protein Assay Kit (Thermo Fisher Scientific, Cat# 23225). Equal amounts of protein were separated by SDS-PAGE and transferred onto PVDF membranes using standard protocols. Membranes were blocked and incubated with the following primary antibodies: Anti-Nrf2 rabbit monoclonal antibody (Thermo Fisher Scientific, Cat# 12721), Anti-LAMINB1 rabbit monoclonal antibody (Cell Signaling Technology, Cat# 12586S) Anti-HSP90 rabbit monoclonal antibody (Cell Signaling Technology, Cat# 4874S). After washing, membranes were incubated with goat anti-rabbit HRP-conjugated secondary antibody (Thermo Fisher Scientific, Cat# 32460). Signals were detected using SuperSignal West Pico Chemiluminescent Substrate (Thermo Fisher Scientific, Cat# 34580), and images were acquired using an iBright CL1000 imaging system (Thermo Fisher Scientific).

### Nrf2 activity assays

Splenic DCs were isolated from mice fed either an n-6 or n-3 rich diet using magnetic purification (Pan DC Isolation Kit, Miltenyi Biotec). Nuclear and cytoplasmic fractions were prepared using the NE-PER Nuclear and Cytoplasmic Extraction Kit (Thermo Fisher Scientific, Cat# 78833) according to the manufacturer’s instructions. Protein concentrations of nuclear extracts were determined using the BCA Protein Assay Kit (Thermo Fisher Scientific, Cat# 23225). Equal amounts of nuclear protein were analyzed for NRF2 DNA-binding activity using the TransAM NRF2 DNA-binding ELISA kit (Active Motif, Cat# 50296), performed following the manufacturer’s protocol. The absorbance was measured at 450 nm using a SpectraMax iD3 instrument (Molecular Devices).

### Experimental Ovarian Cancer Model

Female C57BL/6J mice were fed either an n-6 or n-3 rich diet for 8 weeks prior to tumor challenge. To model metastatic ovarian cancer, mice were injected intraperitoneally (i.p.) with PPNM cells — a transplantable, genetically engineered model of high-grade tubo-ovarian carcinoma (*Trp53*^−/−R172H^*Pten*^−/−^*Nf1*^−/−^*Myc*^OE^) generously provided by Dr. Robert Weinberg (Whitehead Institute) under a material transfer agreement (MTA)^36^. PPNM cells were regularly tested for mycoplasma contamination and maintained under prophylactic Plasmocin supplementation (InvivoGen, Cat# ant-mpp) to prevent infection. For tumor cell inoculation, PPNM cells were suspended in PBS mixed 1:1 with Matrigel matrix (Corning, Cat# 47743–716). Mice received i.p. injections of 1 × 10^6^ cells in a total volume of 200 μl. Metastatic progression, ascites accumulation, and host survival were monitored longitudinally. Tumor burden in the peritoneal cavity was quantified by live bioluminescence imaging. Mice were injected i.p. with VivoGlo Luciferin (2 mg per mouse; Promega, Cat# P1042) and imaged using a Xenogen IVIS Spectrum system at the Weill Cornell Research Animal Resource Center. Bioluminescent signals were recorded and analyzed to assess tumor burden over time.

### DC-based chemo-immunotherapy

Female C57BL/6J mice were maintained on either an n-6 or n-3-fatty acid-rich diet for 8 weeks prior to tumor challenge. Mice were then inoculated intraperitoneally (i.p.) with 1 × 10^6^ PPNM tumor cells. Beginning 14 days post-tumor implantation, mice received cisplatin chemotherapy (5 mg/kg, i.p.) or saline control every 7 days for a total of two doses. For DC-based immunotherapy, starting 15 days post-tumor implantation, mice received either BMDCs (3 × 10^6^ cells/mouse, i.p.) or PBS control every 7 days for two doses, 24 hours post cisplatin or vehicle administration. For adoptive immunotherapy preparations, BMDCs were isolated from C57BL/6J mice fed an n-6 or n-3 PUFA-enriched diet for 8 weeks, following the protocol outlined in the Primary Cell Isolation and Analysis section. Briefly, BMDCs (3 × 10^6^ cells/plate) were cultured in 10 mL complete RPMI medium and stimulated with LPS (100 ng/mL) for 6 hours in bacteriological petri dishes. After stimulation, BMDCs were washed twice with PBS, counted, and prepared for injection. Mice from all experimental groups were monitored regularly for tumor growth, disease progression, and overall survival rates. Long-term survivors (>120 days tumor-free) that had previously received DC-based chemoimmunotherapy were subjected to tumor rechallenge. For this, mice were rechallenged i.p. with 1 × 10^6^ PPNM cells/mouse in PBS mixed 1:1 with Matrigel matrix to assess long-term immune protection.

### In vivo T cell depletion

Long-term survivor mice (>120 days tumor-free) following secondary tumor rechallenge were randomized into two groups: T cell depletion group: Treated with CD4-depleting antibody (Clone: GK1.5, BioXCell, Cat# BP0003–1) and CD8-depleting antibody (Clone: 2.43, BioXCell, Cat# BP0061). Control group: Received IgG2b isotype control (Clone: LTF-2, BioXCell, Cat# BP0090). Antibodies were administered every 3 days for 2 weeks before the third tumor rechallenge. Mice were then injected intraperitoneally (i.p.) with PPNM tumor cells (1 × 10^6^ cells/mouse in PBS mixed 1:1 with Matrigel matrix). T cell depletion therapy continued until the survival endpoint was reached.

### Statistical Analyses

All statistical analyses were performed using the GraphPad Prism software (version 10.0.3). Comparisons between two groups were assessed using unpaired two-tailed Student’s *t*-test. Multiple comparisons were assessed by one-way ANOVA including Tukey multiple comparisons test or two-way ANOVA with Šidák’s multiple comparison test with single pooled variance. Host survival rates were compared using the log-rank (Mantel–Cox) test. Data are presented as the mean ± s.e.m. Exact *P* values are shown, and values greater than 0.05 were considered statistically significant. The data shown are representative from single experiments with three or more biological replicates unless otherwise stated in the figure legends. Experiments were carried out at least three independent times with similar results, unless otherwise stated in the figure legends.

## Supplementary Material

Supplementary Files

This is a list of supplementary files associated with this preprint. Click to download.

- SuppTable1.docx
- SuppTable2.docx
- Suppltable3Fattyacidprofiles.xlsx
- SuppTable4DEGUpdown.xlsx
- SuppFigs.pdf

**Figure S1. Flow cytometry gating strategy and proportion of splenic immune cell subsets in mice under the different diets. A)** Representative gating workflow used to identify major splenic immune cell subsets. B) Mice were maintained on n-6 or n-3 PUFA-enriched diets for 9–10 weeks (n = 4 per group). Spleens were harvested, processed into single-cell suspensions, and stained for flow cytometric analysis of major immune cell populations. Bar graphs show the relative proportions of splenic immune subsets, including B cells, NK cells, macrophages, monocytes, neutrophils, dendritic cells, and T cells. Data are presented as mean ± SEM. Statistical significance was assessed using an unpaired two-tailed Student’s t-test. *P < 0.05, **P < 0.01

**Figure S2. Dietary PUFAs modulate co-stimulatory and MHC-I molecule expression in splenic DC subsets.** Mice were maintained on n-6 or n-3 PUFA-enriched diets for 9–10 weeks (n = 5 per group). DCs were isolated from spleen and stimulated with LPS (100 ng/mL) or vehicle control for 6 hours. Cells were then stained for surface expression of CD40, CD80, CD86, and MHC class I. Representative histograms and bar graphs depict expression levels of the indicated markers on: (A) conventional type 1 DCs (cDC1; CD11c^+^ MHC-II^+^ CD8α^+^) and (B) conventional type 2 DCs (cDC2; CD11c^+^ MHC-II^+^ CD11b^+^). Data are presented as mean ± SEM and represent at least two independent experiments. Statistical significance was assessed using one-way ANOVA followed by Tukey’s multiple comparisons test: *P < 0.05, **P < 0.01, ***P < 0.001, ****P < 0.0001.

**Figure S3**. **Effects of ex vivo exposure to defined n-6 and n-3 fatty acids**. Splenic dendritic cells (spDCs) isolated from chow diet–fed mice were treated ex vivo with the indicated purified n-6 (linoleic acid (LA), Adrenic acid (AA) alone or in combination) or n-3 (eicosapentaenoic acid (EPA), docosahexaenoic acid (DHA) alone or in combination) as described in the Methods. (A) Representative flow cytometry histograms showing CTV dilution in proliferating CD8^+^ T cells co-cultured with fatty acid–treated spDCs. (B) Quantification of T cell proliferation parameters, including percent divided and proliferation index. Data represent at least two independent experiments with comparable results and are presented as mean ± SEM. Statistical significance was determined by one-way ANOVA followed by Tukey’s multiple-comparisons test: P < 0.05.

**Figure S4. Heat map of Nrf2-regulated genes differentially expressed in splenic DCs according to diet.** Heat maps show expression profiles of Nrf2 target genes in spDCs isolated from mice fed n-3 versus n-6 PUFA-enriched diets (n = 5 per group). Genes marked with an asterisk represent those significantly upregulated in n-3–conditioned spDCs that are implicated in glutathione biosynthesis and cellular detoxification (***Gss***, ***Gsr***, ***Shmt2***, ***Mgst1***, ***Gstp1***, ***Gstm5***, ***Gstt2***, and ***Gsta4***), as well as canonical Nrf2 targets involved in redox homeostasis (***Nqo1***, ***Prdx1***, ***Sod3***, ***Gpx1***, and ***Gpx3***). Data reflect relative mRNA expression levels from RNA-seq analysis.

**Figure S5. Glutathione–related metabolites in splenic DCs isolated from mice under PUFA-enriched diets.** spDCs were isolated from mice fed n-3 (n = 7) or n-6 (n = 8) PUFA-enriched diets. Cells were pooled by group, and targeted polar metabolomic profiling was performed by LC–MS/MS. Bar graphs depict fold changes in the relative abundance of metabolites involved in glutathione biosynthesis and redox regulation, including L-methionine, S-adenosylmethionine, S-adenosylhomocysteine, L-serine, L-glutamine, L-cystathionine, L-cystine, L-proline, glycine, acetyl-CoA, reduced glutathione (GSH), and oxidized glutathione (GSSG). Metabolite levels were normalized to cell number prior to analysis.

**Figure S6: Expression of glutathione-related amino acid transporters in splenic DCs from PUFA-fed mice:** RNA-seq data from spDCs isolated from mice receiving either an n-3 or n-6 PUFA-enriched diet (n = 5 per group) are presented as log-normalized counts for transporters associated with the uptake of cystine, cysteine/serine, glutamate, glutamine, glycine, and methionine/glutamine. Statistical significance was determined using unpaired two-tailed Student’s *t*-test: *P < 0.05, **P < 0.01, ***P < 0.001, ****P < 0.0001.

**Figure S7. Dietary PUFAs shape the transcriptomic and functional profile of bone marrow–derived dendritic cells (BMDCs)**. Mice were maintained on n-6 or n-3 PUFA-enriched diets for 9–10 weeks. Bone marrow cells were isolated and differentiated with recombinant GM-CSF to generate BMDCs. CD11c^+^MHC-II^+^ BMDCs were sorted and analyzed as follows: (A) Ingenuity Pathway Analysis (IPA) of RNA-seq data identifying top significantly altered biological processes in BMDCs from n-6 vs. n-3 diet–fed mice (n = 5 per group). (B–C) Total intracellular GSH levels quantified using a Glutathione Assay Kit in (B) untreated or (C) LPS-stimulated BMDCs from each dietary group (n = 3 per group). (D–G) BMDCs from indicated diets were pulsed with OVA and co-cultured with OT-II (CD4^+^) or OT-I (CD8^+^) T cells for 72 h. (D, F) Representative CTV dilution histograms. (E, G) Quantification of T cell proliferation shown as percent divided and division index for OT-II (E) and OT-I (G) T cells (n = 4 per group). (B–G) Data are representative of at least two independent experiments and are presented as mean ± SEM. Statistical significance was assessed using an unpaired two-tailed Student’s t-test: *P < 0.05, **P < 0.01

## Significance

This study identifies dietary PUFAs as critical regulators of DC function via the Nrf2–ferroptosis axis. The findings link nutritional lipid intake to DC competence and therapeutic tumor control, uncovering a dietdirected mechanism that may be harnessed to enhance anti-cancer immunity.
